# Nuclear functions of mammalian MicroRNAs in gene regulation, immunity and cancer

**DOI:** 10.1186/s12943-018-0765-5

**Published:** 2018-02-22

**Authors:** Hongyu Liu, Cheng Lei, Qin He, Zou Pan, Desheng Xiao, Yongguang Tao

**Affiliations:** 1 Key Laboratory of Carcinogenesis and Cancer Invasion, Ministry of Education, Xiangya Hospital, Central South University, 87 Xiangya Road, Changsha, Hunan 410008 China; 2 Key Laboratory of Carcinogenesis, Ministry of Education, Cancer Research Institute, School of Basic Medicine, Central South University, 110 Xiangya Road, Changsha, Hunan 410078 China; 3 Department of Pathology, Xiangya Hospital, Central South University, Changsha, Hunan 410078 China; 4 Department of Thoracic Surgery, Second Xiangya Hospital, Central South University, Changsha, China

**Keywords:** microRNA, Nucleus, PTGS, TGS, TGA, Cancer, Immunity, Metastasis, Invasion

## Abstract

MicroRNAs (miRNAs) are endogenous non-coding RNAs that contain approximately 22 nucleotides. They serve as key regulators in various biological processes and their dysregulation is implicated in many diseases including cancer and autoimmune disorders. It has been well established that the maturation of miRNAs occurs in the cytoplasm and miRNAs exert post-transcriptional gene silencing (PTGS) via RNA-induced silencing complex (RISC) pathway in the cytoplasm. However, numerous studies reaffirm the existence of mature miRNA in the nucleus, and nucleus-cytoplasm transport mechanism has also been illustrated. Moreover, active regulatory functions of nuclear miRNAs were found including PTGS, transcriptional gene silencing (TGS), and transcriptional gene activation (TGA), in which miRNAs bind nascent RNA transcripts, gene promoter regions or enhancer regions and exert further effects via epigenetic pathways. Based on existing interaction rules, some miRNA binding sites prediction software tools are developed, which are evaluated in this article. In addition, we attempt to explore and review the nuclear functions of miRNA in immunity, tumorigenesis and invasiveness of tumor. As a non-canonical aspect of miRNA action, nuclear miRNAs supplement miRNA regulatory networks and could be applied in miRNA based therapies.

MicroRNA (miRNA) is a group of small non-coding RNA that plays significant roles in multiple metabolic processes. Since its discovery in 1993 [1], numerous studies have postulated and established a set of theories concerning miRNA biogenesis and functions, with cross-species researches initially focusing on translational repression in cytoplasm. After transcription, cleavage, and processing, mature miRNA is transported from the nucleus to cytoplasm to be loaded into RNA induced silencing complex (RISC). MiRNA base-pairs with the mRNA, mediating mRNA decay or detachment of ribosomes. In addition to potent inhibitory functions in the cytoplasm, in 2008, research demonstrated that the introduction of miR-373 and its precursor (pre-miR-373) induced the expression of cold-shock domain-containing protein C2 (CSDC2) and E-cadherin [2]. This is attributed to the sequence complementarity of miR-373 and promoters of those genes. In 2004, another member of small non-coding RNA, small interfering RNA (siRNA) was observed to inhibit the transcription of elongation factor 1alpha (EF1A) though promoter interaction [3]. Following these, research interest in promoter-targeting siRNA has increased substantially [4]. With the introduction of newly developed techniques like microarray and RNA-seq, numerous mature miRNAs were found enriched in nucleus, which demonstrates that nuclear miRNAs are more prevalent than what we had thought. Meanwhile, several models elaborating transcriptional gene silencing (TGS) and transcriptional gene activation (TGA) were established involving promoter interaction, non-coding RNA binding, RNA-DNA triplex and enhancer interaction. In addition, recently, several proteins that mediate nucleus-cytoplasm shuttling of key participants in RNA interference (RNAi) were confirmed [5].

This review summarizes nuclear miRNA’s evidence of existence, prevailing models of nuclear miRNA functional mechanisms, practical prediction software tools and associated applications in immunity and cancer. Generally, although different in origin and maturation processes, mature miRNA and siRNA are chemically the same and function similarly when loaded into RISC [6]. Hereby, several researches utilizing siRNA are quoted as analogous examples.

## Canonical biogenesis and functions of MicroRNAs

Canonically, the biogenesis of miRNA is a multi-step process involving both nuclear and cytoplasmic machineries. First, the miRNA gene is transcribed by RNA polymerase II to produce a long pri-miRNA [7], which is then bound to the micro-processing complex consisting of RNA binding protein DGCR8 and the RNase III Drosha [8]. Drosha initiates miRNA maturation by cleaving the pri-miRNA to form the hairpin-structured pre-miRNA [9]. Then the pre-miRNA produced is exported to the cytoplasm via Exportin 5 where maturation is completed [10]. In the cytoplasm, pre-miRNA is recognized with its characteristic 2-nt 3′ overhang by Dicer [11], which cleaves off the terminal loop of the hairpin of the pre-miRNA and generates a miRNA duplex. The protein TRBP stabilizes Dicer and chaperones it with dicing functions [12]. Finally, the miRNA duplex is accommodated and unwound by cytoplasmic Argonaute protein (Ago). One strand is retained to form the functional miRNA-induced silencing complex (miRISC), while the other strand is degraded [13].

**Fig. 1:**
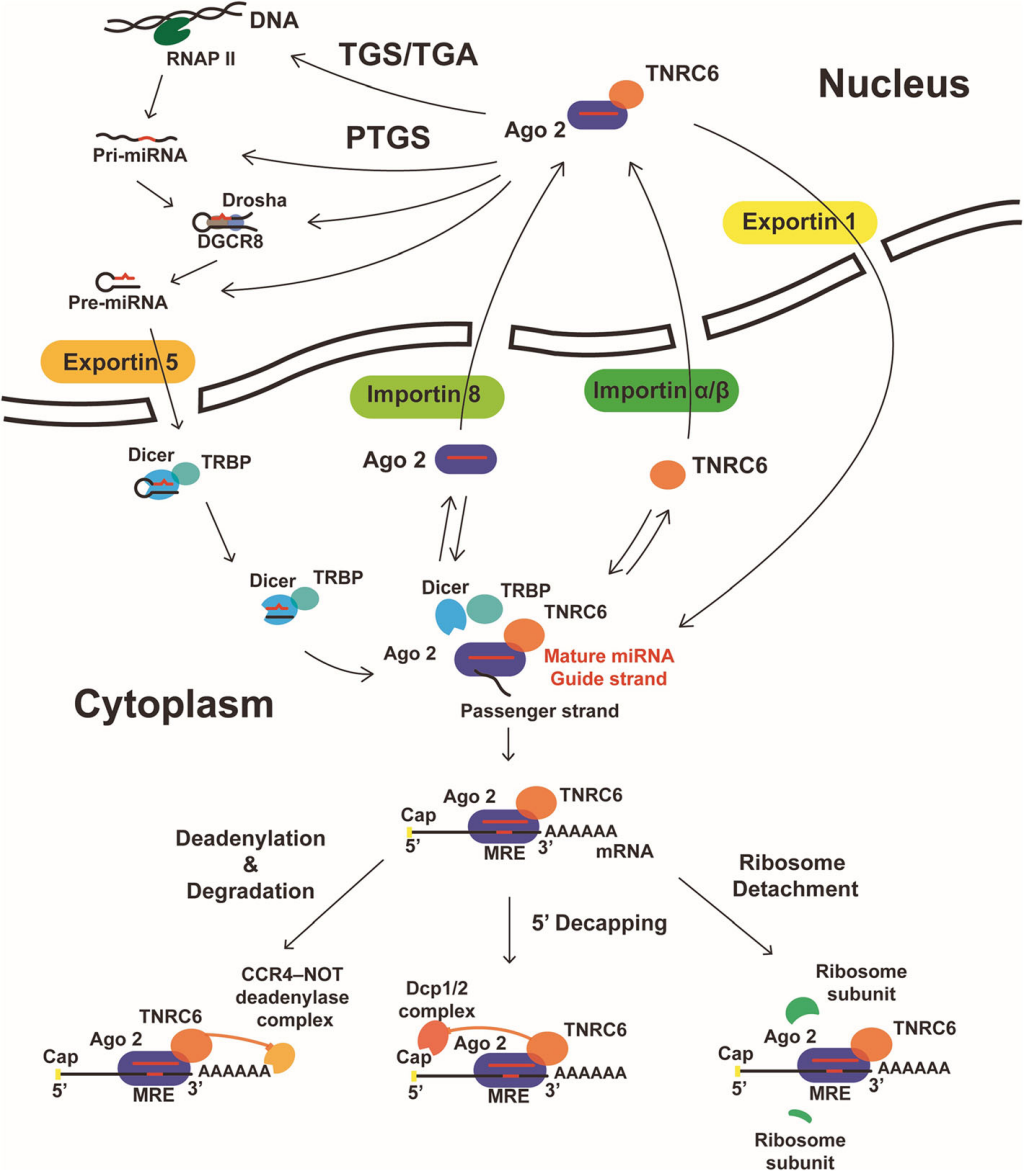
MiRNA biogenesis, function in cytoplasm and nucleus-cytoplasm transport. The biogenesis of miRNA takes several steps. First, pri-miRNA is transcribed via RNA pol II and then cleaved into pre-miRNA by Drosha with DGCR8. Guided by Exportin 5, pre-miRNA is exported to the cytoplasm, where Dicer cuts the stem-loop structure into a double-stranded RNA. The double-stranded RNA formed is then loaded onto Ago 2. One is the mature miRNA, also known as the guide strand. The other is the passenger RNA, which is quickly degraded. Mature miRNA functions both in the cytoplasm and in the nucleus. Core RISC, the major cellular post-transcriptional silencing machinery, is composed of miRNA, Ago 2 and trinucleotide repeat containing 6 (TNRC6). MiRNA recognizes and binds the miRNA response element (MRE) on the mRNA. In the cytoplasm, there are three pathways for miRNAs to exert post-transcriptional silencing: 1) Ago2 recruits TNRC6, which then recruits CCR4-NOT deadenylase complex. This complex leads to deadenylation and degradation of mRNA. 2) TNRC6 recruits Dcp 1/2 decapping complex which cleaves 5′ cap of mRNA and reduces mRNA stability. 3) With binding of Ago 2, mRNA is rendered inaccessible for ribosome attachment and function, which inhibits the translation process. Meanwhile, Ago 2 with mature miRNA is imported into the nucleus via Importin 8 and TNRC6 via Importin β. In the nucleus, RISC is assembled and functions in the transcriptional or post-transcriptional pathway. The nuclear RISC can be transported into cytoplasm via Exportin1 (also referred to as XPO1 or chromosomal region maintenance 1 (CRM1)), which makes transport between the cytoplasm and the nucleus bidirectional. RNAP II, RNA polymerase II; TGS, transcriptional gene silencing; TGA, transcriptional gene activation; PTGS, post-transcriptional gene silencing; Ago2, Argonaute 2; TNRC6, trinucleotide repeat containing 6; TRBP, TAR RNA binding protein; MRE, microRNA response element

Post-transcriptional gene silencing (PTGS) in cytoplasm is the classic function mediated by miRNA via miRISC. For example, miR-139-5p and miR-144 are able to reduce the expression of TET2 and TET3 on both mRNA and protein level [14]. The first step of PTGS is recognition. Some basic principles of base-paring include canonical Watson-Crick A-U, G-C pairing and non-canonical G-U pairing. There is a special sequence on the target mRNA for miRNA recognition and binding called miRNA response element (MRE). The MRE is mostly located at the 3’-UTR of the mRNA, just like TET2 and TET3 mRNA [14]. But some studies suggest that it also occurs in 5’-UTR and even in the protein-coding sequences [15–18]. For mammals, the base-pairing is always imperfect. One example is the base-pairing between let-7 and lin-41. There are two MREs in lin-41, nevertheless, neither of which has perfect complementarity with the 5′-end of let-70 [15]. In addition, recent studies pointed out that translation will be blocked via three pathways: (i) Deadenylation and degradation mediated by CAF1/CCR4/NOT1 complex; (ii) 5′-decapping facilitated by Dcp1/2 decapping complex; (iii) Ribosome detachment from target mRNA (Fig. 1).

**Fig. 2:**
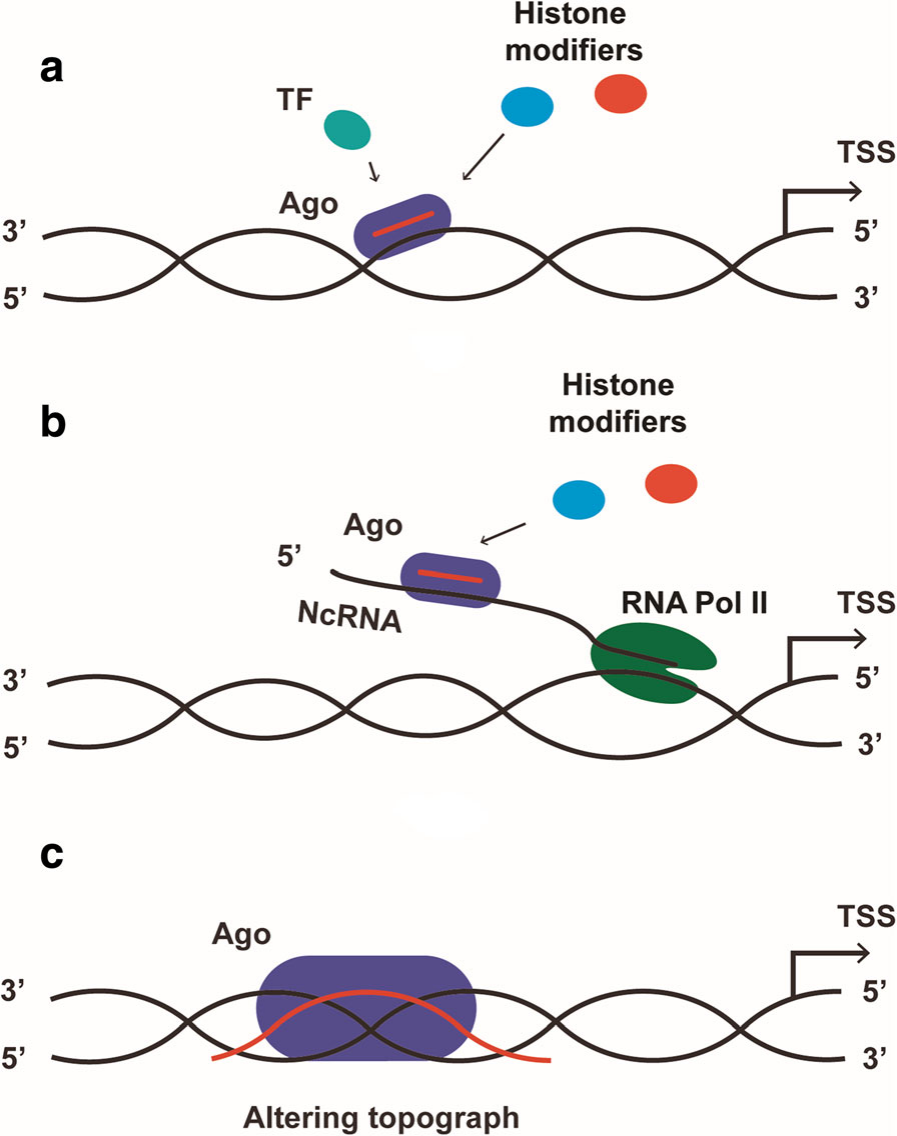
MicroRNA-promoter interaction mechanisms. There are three models for microRNA-promoter interaction, all of which underscore the importance of Ago protein, though there are some conflicting views on whether Ago1 or Ago2 is involved. The resultant interaction effect can be either transcriptional activation or transcriptional suppression. In most cases, the RNA-DNA and the RNA-RNA hybrid model rely on histone modifiers and the recruitment of RNA polymerase II, while whether microRNA can mediate DNA methylation remains dubious. Moreover, in the RNA-DNA-DNA triplex model, the interaction is not related to epigenetic modification. **a** In the RNA-DNA model, microRNA-Ago complex directly targets to one of the DNA strands which always contains the TATA box motif or transcription factor binding sites when this region is in open configuration for transcription. Then some transcription factors and/or epigenetic modifiers are recruited to the promoter region leading to RNA polymerase II recruitment and/or epigenetic modification. **b** The RNA-RNA model is related to the non-coding transcripts derived from the promoter region. Either sense or antisense transcript can be a target for microRNA-Ago complex, serving as a scaffold for microRNA-Ago complex and recruiting the histone modifiers and/or transcription factors. **c** In the RNA-DNA triplex model, microRNAs form triple-helical structures with DNA, altering the topography of the chromatin, which is rendered either more accessible or inaccessible to distinct transcription factors. TF, transcription factor; ncRNA, non-coding RNA; RNA pol II, RNA polymerase II; TSS, transcription start site; Ago, Argonaute 1 or Argonaute 2; Histone modifiers, such as histone methyltransferase enhancer of zeste homolog 2 (EZH2) and euchromatic histone lysine methyltransferase 2 (EHMT2)

## Evidence of MicroRNA in the nucleus

Accumulating evidence suggests that there are miRNAs in the nucleus, though it is putative that miRNA remains in the cytoplasm after its biogenesis [19–24]. Since microRNA was first identified in Hela cell nucleus [25], the existence of nuclear mature miRNAs has been further supported by several studies differentiating microRNA profiles into cytoplasmic and nuclear fractions. With high-throughput profiling technologies such as microarray and deep sequencing, hundreds of nuclei-enriched microRNAs have been identified in a variety of cell lines (Table 1) [26–32]. Some of these results are reaffirmed with Northern Blot, RT-qPCR, RT-PCR and in situ hybridization (ISH) to eliminate precursor signals. It is interesting to note that while nuclear localization of microRNA is a relatively general phenomenon, the microRNA profile in the nucleus varies across tissue types. For example, the nuclear enrichment of human miR-29b has been reported in HeLa cells [33] but not in other cell lines [28]. Notably, the miRNA nuclear-cytoplasmic ratio is identified as a significant feature to distinguish three different breast cancer cell lines [31]. In addition, a web-accessible database RNALocate (http://www.rna-society.org/rnalocate/) provides RNA subcellular localization information which was manually obtained from articles published in the PubMed database before May 2016. Some miRNAs with a nuclear localization can be found in RNALocate [34].

**Table 1:** High-throughput profiling of nuclear microRNAs

Cell line	Method	Result	Year
L6 rat myoblast (nucleolus)	Microarray	One third of the detected miRNAs exhibited nucleolar expression at least as high as those observed in the cytoplasm	2009 [26]
	In situ hybridization	MiR-351, miR-494, miR-664, miR-1, miR-206 are concentrated in the nucleolus MiR-199a-3p, miR-21, miR-125a-5p, and let-7a are more concentrated in the nucleoplasm and cytoplasm	
	RT-qPCR	MiR-351, miR-1, miR-206, miR-21 are concentrated in the nucleolus MiR-494, miR-664 are more concentrated in the nucleoplasm and cytoplasm	
Human nasopharyngeal carcinoma (NPC) 5-8F cell line	Deep sequencing	Among 339 nuclear and 324 cytoplasmic miRNAs, 300 of them overlap	2010 [27]
HCT116 human colorectal carcinoma cell	Microarray	The overall average of nuclear ratio of miRNAs is 0.471 ± 0.15	2010 [28]
	RT-PCR	MiR-16, miR-19b, miR-200b, miR-222, miR-29b, miR-29c are highly expressed in the nucleus	
	Northern blot	MiR-19b, miR-195 are highly expressed in the nucleus	
Human monocytic leukemia cell line (THP-1)	Deep sequencing	MiR-16, miR-15b and miR-374b are all over 2-fold enriched in the nucleus; Nuclear/cytoplasmic ratios of 16 microRNAs are over 1.	2010 [134]
	Northern blot	MiR-15 (both a and b) and miR-16 are over 2-fold enriched in the nucleus	
C57BL/6 J mouse liver cell	Microarray	MiR-709, miR-805, miR-690 and miR-122 are enriched in the nucleus	2012 [49]
	RT-qPCR	MiR-709, miR-690 and miR-30e are highly expressed in the nucleus	
The human breast cancer cell MDA-MB-231	High-throughput sequencing	Almost one-fifth of nuclear small RNAs are annotated as piRNAs	2012 [31]
The human breast cancer cell line MCF-7(noninvasive breast cancer cells), MDA-MB-231(invasive breast cancer cell) and the human mammary epithelial cell line MCF-10A (normal breast cells)	Microarray	Nuclear/cytoplasmic ratios of numerous miRNAs vary considerably across different cell lines	
Rat primary cortical neuron	Microarray	87 (32.6%) miRNAs are dominant in the cytoplasm, while only (1.5%) miR-133b*, miR-365*, miR-328a* and miR-92a are in the nucleus	2013 [29]
	Deep sequencing	MiR-143 and miR-126* are enriched in the nucleus	
	Northern blot	MiR-25, miR-92a, miR-27a, miR-92b are highly expressed in the nucleus MiR-138, miR-19b are highly expressed in the cytoplasm	
	RT-qPCR	MiR-25 and miR-92a are highly expressed in the nucleus	
	Fluorescence in situ hybridization	MiR-25, miR-92 are highly expressed in the nucleus MiR-9 is highly expressed in the cytoplasm	
HeLa cell (nucleolus)	RT-qPCR array	11 miRNAs are highly expressed in the nucleolus	2013 [30]
HeLa cell, lung cancer cell H1299, liver cancer cell Huh7, RPE cells, human adult fibroblast AG06858 and primary mouse adult fibroblast (nucleolus)	In situ Hybridization	MiR191, miR-484, miR-574-3p and miR-193b are highly expressed in the nucleolus	
Four murine myeloid cell lines (LSK, promyelocyte, myelocyte and granulocyte)	RT-qPCR array	Nuclear/cytoplasmic ratios of miR-706, miR-467a*, miR-709, miR-690, miR-135a* (now miR-135a-1-3p) and miR-142-3p are over 0.1 in one or more cell lines; Nuclear/cytoplasmic ratios of miR-706 and miR-467a* (now miR-467a-3p) are over 1 in promyelocytes;	2014 [32]
	Individual RT-qPCR	MiR-706, miR-709 and miR-690 are enriched in the nucleus; MiR-467a* is enriched only in the nucleus of LSK and promyelocyte	

## Shuttle pathways of RISC components

As with nuclear RNA interference [35], many RISC components have been identified that are functional in the nucleus, including Argonaute 2 (Ago2) and trinucleotide repeat containing 6 (TNRC6, also known as GW182) [36–38]. Importin 8, a member of karyopherin β family, has been proven to be the mediator in the nuclear import process of Ago2. Specifically, Ago2 can only be transported when loaded with miRNA [39, 40]. The subcellular distribution of Ago2 varies across cell and tissue. For example, the nuclei of Hela and HaCaT cells present with a minimal level of Ago2 [41]. With both nuclear localization signal (NLS) and nuclear export signal (NES), TNRC6A can be transported from nucleus to cytoplasm with the assistance of Exportin 1 (XPO1, also referred to as CRM1) [42] and inversely with Importin α/β [43, 44].

The nuclear RISC is quite different from its cytoplasmic counterpart: under fluorescence correlation and cross-correlation spectroscopy, RISC presents as an approximately 158 kDa complex in the nucleus whereas 20-fold larger complex of nearly 3 MDa in the cytoplasm [45].When imported into the nucleus, cytoplasmic cofactors such as Dicer and TRBP are not attached to the RISC [45]. Consistently, recent research demonstrated that Ago2 and TNRC6, the core components of RNAi, are conserved in the nucleus and cytoplasm [46].

Given that miRNA is colocalized with Argonaute 2 (Ago2) and TNRC6 in the nucleus and cytoplasm, it is hypothetical that RISC associated proteins serve as not only the executors of RNAi but also agents in the miRNA nuclear import [42] (Fig. 1). Ago2 loaded with miRNA can be transported into nucleus with the mediation of Importin 8, while TNRC6 can be independently transported via Importin α/β. Ago2 and TNRC bind together in the nucleus carrying miRNA, forming a complex, which can be exported via XPO1. This explains why miRNA accumulates in the nucleus with reduction of XPO1 in the previous study [47].

## Functions of nuclear MicroRNAs

### Regulation of RNAs

It has long been observed that nuclear RISC could mediate post-transcriptional gene silencing (PTGS) with miRNA in the nucleus, as well as in cytoplasm. In such a pathway, other endonuclear non-coding RNAs or miRNA precursors could be degraded. In 2005, researchers efficiently degraded nuclear-localized 7SK snRNA with transfection of perfectly complementary siRNA [35]. Long non-coding RNA (lncRNA), a group of non-coding RNAs over 200 nucleotide in length, is also demonstrated to be subject to endonuclear PTGS control. For example, metastasis associated lung adenocarcinoma transcript 1 (MALAT1) is a target of miR-9 in an Ago2-dependent manner in the nucleus [48]. In the nucleus, the maturation of miR-15a/16–1 is inhibited by miR-709 through binding between pri-miR-15a/16–1 and miR-709 [49], which suggests that expression and maturation of one miRNA could be subject to another miRNA.

### Transcriptional regulation by interaction with promoters

There are three models for MicroRNA-promoter interaction based on observation of siRNA directed transcriptional regulation [17, 50] (Fig. 2). All of these three models are associated with the Ago protein, although there are some contradictory results as to whether Ago1 or Ago2 is involved [51]. The regulation can be either activation or suppression, and which mode of action occurs is sensitive to the location of target region (such as TATA box motif and CpG island region) and epigenetic status (such as DNA methylation) of the promoter [52]. Moreover, the target region can be far away from the transcription initiation site. For example, the most effective small activating RNA targeting site is located to 1611-bp upstream of the transcription start site (TSS) [53]. Some histone modifiers have been proven to be recruited to the promoter region. Histone methyltransferase euchromatic histone lysine methyltransferase 2 (EHMT2), which suppresses the expression of fumarate hydratase in nasopharyngeal carcinoma [54], is recruited with enhancer of zeste homolog 2 (EZH2) by miR-584-3p in an Ago2-dependent manner to reduce matrix metalloproteinase 14 (MMP-14) expression in gastric cancer [55]. A recently published study indicated that the genome targeting RNA-induced transcriptional activation complex comprises at least three elements: small RNA-loaded Ago2, RHA (a nuclear DNA helicase II), and CTR9 (a component of PAF1 complex that is involved in transcription initiation and elongation). The complex which targets to the p21 promoter interacts with RNA polymerase II to stimulate transcription initiation and induce mRNA elongation, accompanied by ubiquitination of histone H2B in the p21 gene. This ubiquitination is regarded as a prerequisite for H3K4 methylation and acetylation and followed by histone modification [56]. However, along with the complexity of the epigenetic modifications involved in the regulation process [35], whether miRNA can mediate DNA methylation remains dubious [3, 51, 57].

#### RNA-RNA model

In this model, the microRNA-Ago complex directly targets to noncoding transcripts (either sense or antisense) and serves as a molecular scaffold to recruit additional epigenetic factors, altering epigenetic modifications (such as H3K4me3 and H3K27me3) [58–61]. This complex can also induce enrichment of RNA polymerase II at the promoter region [58, 59, 62].

The transcriptional regulation of siRNA can be different in different cellular contexts. For example, the expression of progesterone receptor (PR) is low in MCF7 breast cancer cells, but higher in T47D cells. Promoter-directed siRNA which activates PR expression in MCF7 cells has no effect or even inhibits PR expression in T47D cells [59, 63]. Some antisense transcripts exist within the PR promoter. Solely reduction of the antisense transcript disrupts the effect of siRNA, but basal expression of PR is not changed in MCF7 cells. They also observed the direct interaction between siRNAs and the antisense transcript by biotin labeling. However, the mechanism through which siRNAs are able to activate or inhibit gene expression is poorly understood [59].

However, another model is suggested for miRNA-promoter interaction based on the data of bidirectional gene regulation [62]. At steady state, endogenous p21 contains comparable levels of both sense and antisense transcripts. Decreased antisense RNA induces loss of H3K27me3 and Ago1 at the p21 promoter, thus downregulating the promoter-associated RNA and upregulating the sense RNA. Decreased sense RNA can also alter the epigenetic modifications.

By targeting the promoter, miRNA can also activate another gene that is located at the upstream of the promoter. The underlying mechanism is related to the noncoding RNA overlapping the promoter and gene looping rendering two linearly distant promoter region spatially proximal [60, 64].

#### RNA-DNA hybrid model

MiRNAs guide Ago protein to promoter targets such as TATA-box motif or the regions associated with transcript factors. Then, transcription preinitiation complex (PIC) is formed, altering histone epigenetic modification as well as binding of transcript factors. Small RNAs might interact with single-stranded DNA while the DNA double helix unwinds during transcription initiation [53, 55, 65–67].

#### RNA-DNA triplex model

In this model, purine or pyrimidine rich (> 75%) miRNAs form triple-helical structures with purine-rich duplex DNA via Hoogsteen or reverse Hoogsteen interaction in the major groove of the duplex DNA. This interaction may alter the DNA topography and allow binding of transcription factors, resulting in transcriptional activation or suppression [68, 69]. Paugh et al. developed an algorithm (Trident) to search for potential triplex-formation sites. Meanwhile, they detected a transient interaction between hsa-miR-5p (a miRNA rich in purine content) with a double-stranded DNA (an identified Hoogsteen binding site screened in genome) in vitro via some methods that are more sensitive and effective in detection of stable triplexes than electrophoretic mobility shift assay (EMSA). Moreover, in primary leukemia cells, largely, the expression of miRNAs that are predicted by Trident are positively correlated with the expression of their target [68]. However, as few evidence exists for triplex formation of miRNAs in vivo [69, 70], this model still needs to be validated upon further study.

### MicroRNAs serve as enhancer trigger in the nucleus

Enhancers are genomic cis-regulatory elements able to upregulate gene transcription. Some markers of enhancer including H3K27ac are found in the microRNA genes (*MIR*s), which means some *MIR*s and enhancers overlap. Some miRNAs, including miR-26a-1, miR-339, miR-3179, miR-24-1, miR-24-2, had been proven to be able to induce expression of neighboring genes [71]. For example, miR-26a-1 gene is surrounded by protein-coding genes *ITGA9*, *CTDSPL*, *VILL* and *PLCD1*. The overexpression of miR-26a-1 will lead to transcriptional activation of *ITGA9* and *VILL* in HEK293T. The activation is disrupted when the seed region of miRNA is deleted or mutated or when the enhancer locus is deleted, which suggests that this function relies on miRNA-enhancer base-pairing. Another miRNA, miR-24-1, also increases the expression of its neighboring genes, *FBP1 *and *FANC*. Increased miR-24-1 will lead to enrichment of RNA polymerase II, p300/CBP, enhancer RNAs, all of which indicate active regulatory functions. Interestingly, ChIP-qPCR demonstrates that there is Ago2 at the enhancer locus. Furthermore, no transcriptional activation of neighboring genes of miR-24-1 will occur if Ago2 is knocked down [71]. So, it could be argued that enhancer activation induced by miRNAs requires Ago2 to function directly at the locus or carry mature miRNA from cytoplasm to the nucleus.

## Similarities and differences between the nuclear and Cytoplasmic functions of MicroRNA

In accordance to those high-throughput profiling results, a large part of miRNAs shuttle between nucleus and cytoplasm, which indicates that they may have both nuclear and cytoplasmic functions. Current knowledge of similarities and differences between miRNA nuclear and cytoplasmic functions are presented as follows (Table 2).

**Table 2:** Similarities and differences between MicroRNA nuclear and cytoplasmic functions

	Cytoplasmic	Nuclear
Regulation Level	Post-transcriptional	Post-transcriptional or Transcriptional
Target	mRNA	Non-coding RNA, pri-miRNA, promoter, enhancer
Mechanism	RNA-RNA hybrid	RNA-RNA hybrid, RNA-DNA hybrid, RNA-DNA triplex
Effect	Silencing	Activation or Silencing

**Table 3:** Nuclear microRNA in cancer

miRNA	Cancer Type	Gene
Tumorigenesis and apoptosis		
miR-423 [73]	Breast cancer	*Human progesterone receptor(PR)*
miR-211 [101]	Mammary carcinoma, B cell lymphoma	*CHOP*
miR-370, miR-1180, and miR-1236 [100]	Bladder cancer	*p21*
miR-205 [86]	Prostate cancer	*IL24 IL32*
miR-124 [102]	Breast and ovarian cancer	*P27*
miR-2478 [135]	Breast cancer	*TGFβ1*
miR-138 [99]	Prostate cancer	*β-catenin*
miR-877 [104]	Bladder cancer	*p16*
miR-6734 [103]	Colon Cancer	*p21*
Metastasis and angiogenesis		
miR-10a [136]	Breast cancer	*Hoxd4*
miR-9 [48]	Non-small-cell lung carcinoma	*MALAT-1*
miR-205 [86]	Prostate cancer	*IL24 IL32*
miR-337 [108]	Neuroblastoma	*Matrix metalloproteinase*
miR-584 [137]	Human neuroblastoma	*Matrix metalloproteinase*
miR-558 [107]	Neuroblastoma	*Heparanase*
miR-337 [109]	Gastric cancer	*Matrix metalloproteinase*
miR-215 [138]	Malignant gliomas	*PCDH9*
miR-2478 [135]	Breast cancer	*TGFβ1*

Both functions are potent in regulation of multiple processes and mediators of miRNA nuclear and cytoplasmic functions partially overlap. Though there is evidence indicating that nuclear RISC is smaller than cytoplasmic RISC [45]. The nuclear localization of Ago2 and TNRC6, as the core RISC, have been proven. Therefore, PTGS can be induced both in nucleus and cytoplasm.

Other than similarities, however, differences are obvious between cytoplasmic and nuclear miRNAs due to their different localization, targets and mechanisms. MiRNAs in cytoplasm only have the access to mature mRNA but not DNA. Hereby, cytoplasmic functions involve only post-transcriptional modification via miRNA-mRNA hybrid interaction. Translation repression is mediated though cleavage, de-adenylation, 5′-decapping or ribosome detachment. Whereas, miRNA in nucleus have the chance to interact with DNA and RNA in nucleoplasm. Therefore, both transcriptional and post-transcriptional regulation exist in nucleus. However, nuclear miRNA shares the same post-transcriptional gene silencing (PTGS) mechanism with cytoplasmic miRNA. Targets in nucleus are endonuclear RNAs including non-coding RNAs and precursors of miRNAs. Transcription regulation is mainly mediated via miRNA-promoter interaction. Three miRNA-promoter interaction models are, as reviewed above, RNA-RNA hybrid, RNA-DNA hybrid and RNA-DNA triplex. For the RNA-RNA hybrid model, miRNA-Ago complex targets non-coding transcripts and recruits epigenetic factors to alter epigenetic modification at the promoter region [59]. MiRNA can also directly target promoter facilitated by transcription preinitiation complex to alter histone modification [56]. In the RNA-DNA triplex model, miRNA forms triple-helical structure with DNA to activate or suppress transcription via altering DNA topography [68, 69]. Recently, enhancer associated mechanism has also been discovered [71]. In conclusion, there are differences of target and mechanisms between nuclear and cytoplasmic functions.

## Nuclear MicroRNA target prediction software tools

Computational prediction of nuclear miRNA target is a critical initial step. Some traditional tools were exploited, while some new ones were developed based on new mechanisms. Target prediction is generally based on sequential characteristics. There are some differences between nuclear and cytoplasmic miRNA target prediction. Here, we summarize some principles of nuclear target recognition and some public software tools.

### Principles of target recognition

The basic principle of microRNA target prediction algorithms is the complement of 5′ end of miRNA and target sequence. As the validation of experimental miRNA-mRNA interaction, several empirical miRNA seed sequence models have been proposed, such as nucleotides from position 2 to 8 in the 5′ end of the miRNA [72]. There is possibly an analogous seed model to be exploited for nuclear miRNA target prediction, as some study shows that the mutation or deletion of seed sequence disrupt the activity of nuclear miRNAs [71, 73].

Additionally, the thermodynamic stability will be measured to evaluate the binding between miRNA and target. A lower free energy indicate a more stable interaction. A widely used software tool to calculate the binding energy is RNAhybrid [74]. There are diverse calculation formula for thermodynamic stability of miRNA-mRNA, miRNA-ssDNA and miRNA-dsDNA interaction, so different algorithms will be used for cytoplasmic miRNA and nuclear miRNA target predicting [68].

Another valuable trait of sequences is their evolutionary conservation. There is more possibility a sequence is vital if it is more conserved. Conserved regions in promoters largely overlap open chromatin regions and TF binding sites [75]. In some prediction software tools, conservation of seed sequences will be measured to prevent some false positive results. However, sequence conservation of the promoter is generally lower than that of gene coding regions. Hereby, non-conserved regions should not be ignored. To score the conservation of the sequences independently is an alternative way to solve this problem [72].

With the development of high-throughput sequencing, some public experimental data can be incorporated to identify the miRNA-target interaction. For example, paired expression profiles of miRNAs and mRNAs have been used to identify functional miRNA-target [76]. Ago binding sequences and nuclear/cytoplasmic localization data from previous deep sequencing experiment have been incorporated for miRNA-promoter interaction prediction [77].

### Public software tools

MicroPIR2 (http://www4a.biotec.or.th/micropir2/) predicts miRNA target of mouse and human promoter region with related genomic and experimental information. The nuclear/cytoplasmic localization and data obtained from some studies and experimentally verified binding sites of Ago proteins are also incorporated into the database [77].

MiRwalk2 (http://zmf.umm.uni-heidelberg.de/apps/zmf/mirwalk2/) provides predicted and validated information of miRNA-target interaction. The binding sites of miRNA can be set to be within all regions of a gene (promoter, 5’-UTR, CDS and 3’-UTR). Results of other 12 miRNA-target prediction programs are also combined and analyzed in miRwalk2 [78, 79].

Trident (http://trident.stjude.org) is a computational algorithm to identify Hoogsteen and reverse Hoogsteen interactions between single-stranded oligonucleotides (microRNA) and double-stranded oligonucleotides (double-stranded DNA). The thermodynamic binding score and heuristic score are measured to evaluate the interaction of miRNA and DNA. Higher heuristic score and lower thermodynamic energy indicate a stronger interaction [63].

Other traditional miRNA target prediction software tools or algorithms can also be used for nuclear miRNA analysis. In order to prevent false positive results, some miRNA target prediction algorithms require conservation of seed sequence and limit the target site in the 3' UTR of mRNA. However, the nuclear miRNAs-targeted promoters are always poorly conserved. There are useful software tools with less limitations or more optional parameters such as TargetS (http://liubioinfolab.org/targetS/mirna.html) [72], RegRNA 2.0 (http://regrna2.mbc.nctu.edu.tw/index.html) [80], and some downloadable applications such as miRanda [81] and RNAhybrid (https://bibiserv.cebitec.uni-bielefeld.de/rnahybrid) [74].

## MicroRNA nuclear functions in immunity

### Granulopoiesis

Some microRNAs have been identified as key modulators of granulopoiesis through detection of the miRNA expression profiles at different stages of hematopoietic differentiation [32, 82, 83]. Several miRNAs predominantly localize in the nucleus in murine myeloid cells, such as miR-223, miR-706, miR-690, miR-709 and miR-467a* [32, 84]. In the cytoplasm of myeloid progenitor cells, miR-223 targets transcription factors MEF2C and NF1A, resulting in increased granulopoiesis. However, in the nucleus, miR-223 also targets the NF1A promoter, recruiting the polycomb repressive complex and increasing the DNA methylation and repressive histone markers [84]. Thus, miR-223 could be an essential determination factor of hematopoietic proliferation and differentiation.

### Cytokine

Nuclear microRNAs have the ability to regulate the activation of immune cells via expression regulation of cellular cytokines. For example, let7i binds to the TATA-box and activates the transcription of interleukin-2 (IL-2) in CD4+ T-lymphocytes [60], which is an early landmark critical in the activation of CD4+ T cells [85]. In prostate carcinoma cells, miR-205 activates IL-24 and IL-32 by targeting their promoters. IL-24 and IL-32 are members of cytokine family both serving as tumor suppressor [86]. IL-24 is also known as melanoma differentiation-associated-7 because of its tumor suppression function. It is mainly expressed and functions in non-hematopoietic tissues as an inducer of cell death [87]. Moreover, IL-32 is a proinflammatory cytokine, which also functions in cell differentiation, activation of NK and NKT cells, maturation and activation of immature DCs and infection control, etc. [88]. Thus, nuclear microRNA may play an important role in the pathway of immune response and signal transduction by targeting the promoter of cytokine genes.

### Inflammation

Two adjacent inflammatory genes, cyclooxygenase-2 (*COX-2*) and phospholipase A2 (*PLA2G4A*) are both activated by miR-589. The targets of miR-589 are located in the promoter of *COX-2*, and gene looping facilitates the interaction between promoters of *COX-2* and *PLA2G4A*. *PLA2G4A* encodes an enzyme which catalyzes hydrolysis of membrane phospholipids to release arachidonic acid. COX-2 catalyzes the conversion of arachidonic acid to prostaglandins, an inflammatory factor which plays an important role in vasodilation [60].

### Asthma

CD44, a transmembrane glycoprotein, plays a vital role in a variety of immune processes including lymphocyte activation, cell proliferation and assembly of inflammatory cells. Recently, some studies [89, 90] demonstrated that CD44 is associated with airway inflammation, particularly asthma, where Li et al. [91] found high expression level of miR-31 in asthma patients. Further study revealed that miR-31 can directly bind to the promoter of *CD44* gene and upregulate its expression. Overexpressed CD44 could further induce the expression of asthma-associated molecules, including IL-6, IL-8 and intercellular adhesion molecule 1 (ICAM), which promote progression of asthma.

## MicroRNA nuclear functions in cancer

Cancer progression is a metabolically dynamic process that involves tumor initiation, promotion, progression, angiogenesis and metastasis. In a classic cytoplasmic manner, numerous studies illustrate the significant role miRNAs play in cancer. For example, In glioma, miR-200c represses tumor growth and metastasis via interaction with moesin (MSN) mRNA [92]. MiR-4260 serves as a oncopromoter in the colorectal cancer by targeting tumor suppressor colorectal mutant cancer protein (MCC) and SMAD4 [93].

Additionally, nuclear miRNAs have been reported to be involved in the transcriptional regulation of a variety of tumor promoter/repressor genes or genes that are cancer-related by acting upon the promoter of the respective gene loci. Intriguingly, other acting mechanisms of miRNA have also been reported [94]. Over the recent years, a burgeoning literature has been focused on nuclear miRNAs and cancer. Despite ongoing challenges, sizable results have been achieved. Here we attempt to review the current progress of this research endeavor (Table 3).

### Tumor initiation, self-sustenance and apoptosis

It is of general consensus that cancer initiation and progression results from aberrant control and integration of growth, differentiation and apoptosis regulatory signals, which could lead to immortalized cell groups capable of self-sustenance and auto-renewal [95]. Apoptosis is an orchestrated and ordered cellular process in which cells experience programmed cell death under physiological and pathological conditions. Defects can occur at any point along apoptotic pathways, leading to malignant transformation of the affected cells, tumor metastasis and drug resistance [96]. Recent researches in nuclear miRNA and transcriptional gene silencing/activation mechanisms have provided insights that could enrich our understanding in tumorigenesis and apoptosis processes.

Studies using chromatin immunoprecipitation and RNA mimicking reveal that miR-423-5p decreases RNA polymerase II occupancy and increases histone H3 lysine 9 dimethylation (H3K9me2) at the progesterone receptor (PR) promoter of human breast cancer cells, indicating a chromatin-level silencing mechanism for the regulation of expression of PR, which mediates endocrinal effects in the development of the mammary gland and breast cancer [97].

MiR-483, an oncogenic intronic miRNA, is reported to bind to the most upstream imprinted insulin-like growth factor 2 (IGF2) gene promoter P2. Ectopic expression of miR-483 induces upregulation of IGF2 expression, as well as an increase in tumor cell proliferation, migration, invasion, and tumor colony formation [98].

Alpha-methylacyl-CoA racemase (AMACR) is highly overexpressed in prostate cancer (PCa) and its transcriptional regulators include various transcription factors and CTNNB1/β-catenin. Studies conducted in vitro in PCacells by Erdmann K et al. [99] revealed that miR-138 indirectly up-regulates AMACR via transcriptional induction of CTNNB1.

Three miRNAs (miR-370, miR-1180 and miR-1236) induce nuclear p21 expression through p21-promoter binding. The expression levels of three miRNAs decrease in bladder cancer tissues compared to healthy controls. Meanwhile, expression of these miRNAs positively correlate with p21 mRNA expression. Overexpression of these three miRNAs inhibits the proliferation of bladder cancer cells mainly by regulating p21 [100].

MiR-211 is a pro-survival microRNA that down-regulates the pro-apoptotic transcription factor C/EBP-homologous protein (CHOP) in a stress and PERK-dependent manner. This permits the cell to prevent premature accumulation of CHOP and rebuild homeostasis prior to apoptotic activation [101]. Studies using functional proteomics demonstrated a RNA-activation function of miR-124 resulting in direct induction of p27 protein expression by binding to and inducing transcription on the p27 promoter region, leading to a subsequent G1 arrest. Ensuing in vivo studies utilizing a xenograft model demonstrated that nanoparticle-mediated delivery of miR-124 could reduce tumor growth and sensitize cells to etoposide to increase apoptosis [102]. Similarly, miR-6734 was found to inhibit the growth of colon cancer cells by up-regulating *p21* gene expression and subsequent induction of cell cycle arrest and apoptosis [103]. The binding site of miR-877-3p was also found on the promoter site of tumor suppressor gene *p16*. *P16* exerts a similar function on inhibiting the proliferation and tumorigenesis of bladder cancer through induction of G1 arrest [104]. P16/INK4a is hypothesized to modulate EPCs senescence with telomerase [105].

Overall, nuclear miRNAs, which mainly act upon the promoter regions, are observed to affect the expression profile of oncogenes, tumor suppressors or other cancer-related genes in the cancer initiation process.

### Metastasis and angiogenesis

Tumor metastasis involves development of genetic and epigenetic alterations in malignant cells, which could result in global dissemination of cancer cells and life-threatening conditions in patients. Angiogenesis in a key step in the metastatic cascade and provides the primary tumor the main route for transport through the vasculature [106]. A number of metastasis regulators have been discovered in recent years that are targets of miRNA endonuclear mechanisms, including matrix metalloproteinase (MMP) [107–109], protocadherin 9 (PCDH9) [110], E-cadherin [2], cold shock domain containing C2 (CSDC2) [2], etc., many of which are involved in the regulation of extracellular matrix (ECM) integrity or effectors of the epithelial–mesenchymal transition.

E-cadherin belongs to cadherin superfamily and functions as adhesion regulator between cells. This gene is considered a tumor repression gene, the loss of which is observed in many malignant tumors. MiR-373 induces expression of E-cadherin and CSDC2 through promoter binding [2]. MiR-205 increases the expression of tumor suppressor genes *IL24* and *IL32* in prostate cancer. Transfection of miR-205 leads to decrease of cell growth, metastasis and invasiveness of prostate cancer cells [86].

Matrix metalloproteinase 14 (MMP-14) is a membrane-anchored MMP that promotes migration and invasiveness in various tumors. Both miR-337-3p and miR-584-5p inhibit the transcription of MMP-14 in human neuroblastoma. Overexpression of both miRNAs leads to decrease of growth, metastasis and angiogenesis of human neuroblastoma in vitro and in vivo [108, 111]. In gastric cancer, miR-337-3p inhibit myeloid zinc finger 1 (MZF1) induced transcriptional activation of MMP-14 through recruiting Ago2 and inducing repressive chromatin remodeling. MiR-337-3p attenuates growth, migration, and angiogenesis of gastric cancer cells in vitro and in vivo [109].

MiR-558 can bind to the promoter of Heparanase (HPSE) and enhance its expression activity in an Ago-1-dependent manner. Heparanase is an endoglycosidase which degrades polymeric heparan sulfate molecules and is associated with migration and invasiveness of tumor, like in choriocarcinoma [112]. Consistently, knockdown and over-expression of miR-558 indicate its positive function on tumorigenesis and aggressiveness in neuroblastoma cells [107].

Protocadherin 9 (PCDH9), a member of cadherin superfamily, facilitates cell-cell adhesion and is downregulated in glioma. MiR-215-5p binds to both promoter and 3’ UTR of PCDH9 and inhibits the expression of PCDH9 in glioma [110].

Metastasis Associated Lung Adenocarcinoma Transcript 1 (MALAT-1), a nuclear long non-coding RNA, plays an important role in the metastasis in non-small-cell lung carcinoma (NSCLC). In previous studies, miR-9 post-transcriptionally regulate MALAT1 in an Ago2-dependent manner in the nucleus [48].

### Application of RNAa in cancer therapeutics

In addition to RNA activation (RNAa) triggered by endogenous miRNAs as previously described [2, 86, 100, 102, 107, 113], RNAa has also been exploited and applied in researches of cancer therapeutics and tumorigenesis. Tumorigenesis is a complex process that often entails underexpression of a variety of tumor suppressor genes involved in multiple signal transduction pathways. RNAa, which is highly specific and poses far less danger to genome integrity than viral vectors, could be utilized to upregulate a cohort of tumor suppressors and thus relieve tumor progression. In vivo and in vitro studies have been conducted with effective tumor inhibitory outcomes [114–119].

P21 (also referred to as cyclin-dependent kinase inhibitor 1 or CDK-interacting protein 1) is a tumor suppressor and an important cell cycle regulator which relays the upstream effect of p53 gene and inhibits cyclin-dependent kinase (CDK). Overexpression of p21 results in inhibition of cell-cycle progression and G1 arrest, thus could potentially repress tumor progress [120]. In vivo and in vitro studies of p21 RNAa have been conducted in researches where inhibition of proliferation in prostate, lung, hepatocellular, pancreatic and bladder cancer cells was observed [118, 121–124]. In another study, hepatocellular carcinoma cells were transfected with dsRNA by liposomes targeting the *Wilms’ tumor 1* (*WT1*) gene promoter, which upregulated WT1, a potent tumor suppressor, and resulted in increased apoptosis of malignant cells [125]. The selected small activating RNA (saRNA) also inhibited the growth, invasion and migration of GC cells by specially reactivating vezatin (VEZT), which resulted in an obvious decrease in the proliferative, invasive and migratory abilities of cancer cells [126]. Moreover, RNAa has been used to promote E-cadherin expression and repress tumor invasion and metastasis in vivo and in vitro in breast, prostate and bladder cancer [116, 127, 128].

Additionally, RNAa has been studied in sensitizing tumor cells to chemotherapy. Innate or acquired resistance to chemotherapy is a formidable challenge that confronts oncologists. Recently, cisplatin nanoparticles co-loaded with miR-375 was developed as a promising treatment for the hepatocellular carcinoma (HCC) based on classic theory of interaction between miRNA and mRNA [129]. Moreover, in one study, saRNA targeting to p21 was transfected in lung cancer cells that are simultaneously treated with cisplatin. Both in vitro and in vivo experimentations showed transfected groups with enhanced chemosensitivity to cisplatin [118], indicating that saRNA based approaches could be potentially applied to relieve chemotherapy resistance under clinical settings.

## Conclusion

Since its discovery in 1993, miRNAs had been assumed as post-transcriptional gene regulators that function by base-pairing with mRNAs to inhibit the translation process. However, in more recent years, it has been demonstrated that miRNA could also play a regulatory role in the nucleus [2, 130]. Meanwhile, shuttling mediators of key proteins in the microRNA pathway have also been found. There is some evidence pointing out that miRNA could direct traditional RISC-mediated post-transcriptional gene silencing (PTGS) activities endonuclearly. MiRNA mainly targets specific nucleotide sequences in the promoter region, recruiting epigenetic remodelers and altering gene expression profiles. MiRNA is also shown to serve as enhancer triggers [66] in the nucleus. Disturbances of nuclear miRNA activities could be implicated in a number of diseases and disorders as well as normal physiological processes in immunity and tumor [60, 88, 98].

Based on different targeting prediction principles, many algorithms have been designed. However, because of the deficiency in sensitivity and specificity, no program is proven superior to the others. Results from 12 different miRNA-target prediction programs are integrated into the miRwalk2 database. This database also provides validated target information by an automated text-mining search in the titles/abstracts of the PubMed articles. As research continues to reveal miRNA regulatory mechanisms, the algorithms will become more sensitive and accurate [78, 79, 131].

However, some early results are not repeatable and even conflict with later results. One example is the hexanucleotide element for miR-29b nuclear entrance [33]. MiR-29a without this element will be retained in cytoplasm. However, enrichment of miR-29b is not observed in other cell lines [28]. One explanation is that localization varies among different cell lines. Another explanation is error due to technique. Purity of nuclear abstraction is hard to guarantee. Recently, a protocol for nucleus-cytoplasm division was developed to solve this problem [132].

Nowadays, miRNA therapy and related researches are in a trend to becoming more and more common, but most of them are based on cytoplasmic functions rather than nuclear functions [133]. Accumulating evidence urge us to consider the yet not-too-familiar effect miRNA exerts on the transcription level. As our understanding of its function and dysregulation deepens, nuclear miRNA promises to have applications in a number of clinical settings in the future [4].
